# Conscious Sedation Methods for Blepharoplasty in Day Surgery

**DOI:** 10.3390/jcm12124099

**Published:** 2023-06-17

**Authors:** Tae-Yul Lee, Han-Jin Bae, Deok-Woo Kim, Too Jae Min

**Affiliations:** 1 Department of Plastic and Reconstructive Surgery, Korea University Ansan Hospital, Ansan-si 15355, Republic of Korea; tylee0919@korea.ac.kr (T.-Y.L.);; 2 Department of Anesthesiology and Pain Medicine, Korea University Ansan Hospital, Ansan-si 15355, Republic of Korea

**Keywords:** blepharoplasty, dexmedetomidine, conscious sedation, hematoma, aesthetic surgery

## Abstract

Midazolam and fentanyl, in combination, are the most commonly used medications for conscious sedation in day aesthetic surgeries. Dexmedetomidine is popularly used in the sedation protocol of our hospital due to its reduced respiratory depression. However, its sedation benefits in facial aesthetic surgeries, like blepharoplasty, have not been well-evaluated. We retrospectively compared individuals sedated with midazolam and fentanyl bolus injection (N = 137) and those sedated with dexmedetomidine infusion (N = 113) to determine which is more suitable for blepharoplasty with a mid-cheek lift. The total amount of local anesthetic (*p <* 0.001), postoperative pain (*p =* 0.004), ketoprofen administration (*p =* 0.028), and the number of hypoxia episodes (*p <* 0.001) and intraoperative hypertension (*p* = 0.003) were significantly lower in the dexmedetomidine group. Hypoxia severity (*p <* 0.001) and minor hematoma formation (*p =* 0.007) were also significantly lower in the dexmedetomidine group. Sedation with dexmedetomidine infusion is associated with less hematoma formation than sedation with midazolam and fentanyl bolus pattern due to hemodynamic stability and analgesic effects. Dexmedetomidine infusion may be a good alternate sedative for lower blepharoplasty.

## 1. Introduction

Blepharoplasty is the most popular cosmetic surgery in South Korea, and its demand is increasing yearly worldwide. Anatomically, the eyelids are composed of a very thin dermis and little subcutaneous fat [1]. Opening the eyes and the eyelid shape are profoundly affected by swelling during or after surgery, which could increase the patient’s discomfort, decrease operation satisfaction, and delay recovery to daily life [2]. Blepharoplasty success depends on the minimization of swelling during the intraoperative and immediate postoperative periods.

Insufficient pain management increases blood pressure and the risk of postoperative ecchymosis, hematoma, and swelling [3]. Additionally, hematomas increase the likelihood of intraoperative and immediate postoperative swelling. Hematomas can have negative consequences in facial surgery, leading to scarring and increasing the chances of infection, facial edema, skin hyperpigmentation, neuropraxia, and prolonged recovery [4,5]. Specifically, hematomas can cause long-standing “knots”, contour irregularities, and puckering under the skin. Plastic surgeons must consider sedation that minimizes swelling and hematoma formation.

Several studies have reported that there may be a relationship between the use of nonsteroidal anti-inflammatory drugs (NSAIDs) and the incidence of hematomas [6,7]. Considering that NSAIDs could increase the tendency to bleed, a decrease in the use of NSAIDs may reduce the formation of postoperative hematomas. We reviewed a total of 419 patients who underwent lower blepharoplasty with a mid-cheek lift under sedation anesthesia between 2018 and 2022, to determine which sedation method is more suitable for lower blepharoplasty and a mid-cheek lift.

## 2. Materials and Methods

### 2.1. Materials

The study protocol was approved by our Institutional Review Board (approval number: 2021AS0004). This study was conducted per the principles of the Declaration of Helsinki. We retrospectively reviewed the medical records of 419 patients who underwent lower blepharoplasty with mid-cheek lift at our institution between March 2018 and May 2022. Lower eyelid and midface rejuvenation was performed in patients who wanted to improve infraorbital hollowness, tear trough deformity, and lower lid fat bulging. We evaluated the operation time, intraoperative airway depression, pain visual analog scale (VAS) score, amount of local anesthetic injected, length of stay in the post-anesthesia care unit (PACU), and surgical details using medical charts. Of the 419 patients, those who met the following inclusion criteria were included: (a) primary lower blepharoplasty or midface lift; (b) American Society of Anesthesiologists (ASA) physical status classification ≤ II; (c) no recent drug history of anticoagulants, opioid analgesics, or herbal medicine; and (d) patients who wanted to undergo conscious sedation in day surgery. We obtained informed consent from all patients.

### 2.2. Sedation Regimens

Anesthesiologists performed all of the sedations. The sedation protocol subgroups were categorized as (a) bolus fashion using midazolam with or without fentanyl (N = 137; conventional group) and (b) continuous fashion using dexmedetomidine hydrochloride (N = 113; dexmedetomidine group). Oxygen was supplied through nasal prongs during surgery in both groups. At a flow rate of 2–5 L/min, oxygen was administered, and following the application of an aseptic drape, the sterilized nasal prongs were opened and taped securely in place with the intention of preventing contact with the operative site of the blepharoplasty.

### 2.3. Sedation Protocols

#### 2.3.1. Bolus Fashion Using Midazolam and Fentanyl: The Conventional Method

Patients in the midazolam/fentanyl group received midazolam (0.05 mg/kg or 5 mg) and fentanyl (0.5–1.5 µg/kg), injected as a bolus before the incision. Midazolam was titrated in 1 mg/bolus increments until the patient was visibly relaxed but responsive (maximum midazolam dose: 5 mg). Fentanyl was titrated in 25 µg/bolus injections before surgical incision, and 25 µg/bolus increments were utilized when patients could not tolerate pain or had a VAS score ≥ 4. The interval between the bolus doses was 5 min (maximum fentanyl dose: 1.5 µg/kg). 100 mg of ketoprofen (maximum dose, 300 mg/day) was administered, or more local anesthetics were infiltrated when the maximum fentanyl dose was administered (1.5 µg/kg).

#### 2.3.2. Continuous Fashion Using Dexmedetomidine

Patients in the dexmedetomidine group (Precedex; Pfizer, New York, NY, USA) received a loading dose of dexmedetomidine (1 µg/kg) over 10 min followed by a continuous infusion (0.2–0.7 µg/kg/h). Fentanyl and ketoprofen were not administered, and only dexmedetomidine was used as a sedative. Local anesthetics were infiltrated at the surgical site.

### 2.4. Postoperative Analgesic Regimen

A dose of 100 mg of ketoprofen was administered to patients with a VAS score > 4 for rescue analgesia. This was repeated after 5 min, if necessary (maximum dose of ketoprofen: 300 mg/day).

Meperidine (0.25 mg/kg) was given for rescue analgesia if postoperative pain was not controlled (VAS score ≥ 4), despite the maximum rescue dose of ketoprofen.

### 2.5. Perioperative Monitoring and Definition

Cardiac function (electrocardiogram), noninvasive arterial pressure, and peripheral oxygen saturation (SpO_2_) were monitored. The anesthetic depth was monitored using the cortical activity index (CAI) via the CAI™ Monitoring System (BrainU Inc., Seongnam, Gyeonggi-do, Republic of Korea). The anesthetic depth was maintained at a CAI level between 60–80, which is equivalent to the bispectral index (BIS) sedation range. Notably, 2 L of O_2_ was administered during sedation.

### 2.6. Definition of Variables

The degree of pain was assessed using the VAS, consisting of a 10 cm line, with two end-points representing 0 (no pain) and 10 (worst pain). Hypoxia during surgery was graded as follows: (a) normal, 95–100% SpO_2_; (b) mild hypoxia, 91–94% SpO_2_; (c) moderate hypoxia, 86–90% SpO_2_; and (d) severe hypoxia, ≤85% SpO_2_. Postoperative hematomas were classified as major and minor hematomas. Major hematomas were emergent and required open evacuation by removing sutures and coagulating the offending bleeders. Minor hematomas involved smaller amounts of blood or serum that could be resorbed with a massage or spontaneously without treatment. Hypotension was defined as systolic blood pressure SBP  <  100 mmHg and a decrease of >20% from baseline for more than 5 min. Hypertension was defined as SBP  >  160 mmHg and an increase of >20% from baseline for more than 5 min [8]. The length of PACU stay was defined as arrival to the PACU to the shift to the day-care center.

### 2.7. Surgical Procedures

All surgical procedures were performed under local anesthesia with 1% lidocaine and 1:100,000 epinephrine and conscious sedation. The pretarsal crease, nasojugal groove, and palpebromalar groove were marked preoperatively with the patient in a seated position. An incision was made 2 mm below the ciliary line with a lateral extension. After elevating the skin flap by 5–6 mm, the orbicularis oculi muscle (OOM) was split transversely. The skin-muscle flap was elevated using monopolar electrocautery. Premaxillary space dissection was performed from the arcus marginalis to 2–3 mm below the orbital rim. The periosteum was then resected. A subperiosteal dissection was performed 3–4 mm down to make a periosteum cuff. The palpebral part of the OOM, tear trough ligament, and orbital part of the OOM were sequentially released. When visualizing the levator labii superioris muscle fibers on the floor, the entrance to the premaxillary space was visible. Premaxillary space dissection was performed using periosteal elevators to raise the deep nasolabial and suborbicularis oculi fat areas. Laterally, the orbicularus-retaining ligament was released using monopolar electrocautery. When the dissection was complete, medial and central orbital fat were transposed and placed under the periosteal cuff. The authors used a modified method of the fat preservation technique (fixing the capsulopalpebral fascia to the arcus marginalis) first reported by Mendelson. By fixing the capsulopalpebral fascia to the periosteal cuff, the orbital fat transposes into the periosteal pocket, creating a secure septal tightening effect. Then, the surgeon evaluated the bulging of the lateral fat pad. Excess fat was removed if necessary, followed by plication of the septum to prevent herniation. The elevated deep nasolabial fat was suspended superolaterally and fixed to the arcus marginalis using 4-0 nylon sutures (deep nasolabial fat lifting). The OOM was subsequently suspended and secured to the inner aspect of the lateral orbital rim using a vicryl 5-0 suture (OOM suspension). The lower lid skin was draped over the lower eyelid, and excess skin was marked while the patient looked upward with their mouth open. The excess skin was then excised conservatively, and the incision was repaired using a 7-0 nylon suture.

### 2.8. Statistical Analysis

We used Pearson’s chi-square test or Fisher’s exact test to compare the categorical variables and an independent *t*-test to compare the quantitative variables of the subgroups. We performed all statistical analyses using SPSS version 10 (SPSS Inc., Chicago, IL, USA). The statistical significance was set at *p* < 0.05.

## 3. Results

A total of 250 patients were included in this study, 161 (64.4%) of whom were women. The subjects’ mean age was 57.8 years (range, 23–84 years). On average, the patients were of normal weight with a body mass index of 24.4 kg/m^2^ (range, 17.4–35.5 kg/m^2^). The proportion of ASA class was 12.8% for class I and 87.2% for class II. There were no significant differences between the demographic characteristics of the two groups (Table 1).

The distribution of sedation methods was assessed according to each year (Table 2). There was no significant difference observed between the two groups (*p* = 0.517). The dexmedetomidine group was slightly more frequently utilized during the initial three years, while the conventional group consistently remained in use, with over 60% utilization in the latter two years.

Table 3 shows the differences in surgery-related profiles between the conventional and dexmedetomidine groups. The total amount of local anesthetic was significantly lower (*p <* 0.001), but the total length of the incision and the operation duration were longer in the dexmedetomidine group, although this difference was not significant. The number of incisions was two in both groups.

Hypoxia, hematoma, and intraoperative hypertension events were analyzed to compare the complication-related profiles (Table 4). The number of hypoxia episodes was significantly higher (*p <* 0.001) in the conventional group than in the dexmedetomidine group, and there were only three episodes of hypoxia reported in the dexmedetomidine group. Hypoxia severity (*p <* 0.001) and minor hematoma formation (*p =* 0.007) were also significantly different between the conventional and dexmedetomidine groups. In the dexmedetomidine group, the incidence of mild/moderate hypoxia was observed in only three patients (2.7%), and there were no cases of severe hypoxia. There was one (0.7%) patient with major hematomas in the conventional group. One major hematoma required open evacuation and coagulation of the offending bleeders. Minor hematoma occurred in 16 patients: 2 in the dexmedetomidine group and 14 in the conventional group, indicating a significant difference between the groups. Exploration of minor hematomas was not necessary, and they spontaneously disappeared after compression dressing. Hypertension events were significantly higher in the conventional group compared to the dexmedetomidine group (*p* = 0.003).

To compare the recovery-related profiles, we analyzed the length of PACU stay, pain, nausea, and urinary catheterization (Table 5). The length of PACU stay was significantly longer (*p <* 0.001) in the dexmedetomidine group, while postoperative pain (*p =* 0.004) and analgesic (ketoprofen) administration (*p =* 0.028) were significantly lower. However, postoperative nausea and antiemetic administration rates were not significantly higher in the dexmedetomidine group. Six (4.4%) patients underwent urinary catheterization in the conventional group, while two (1.8%) patients in the dexmedetomidine group did. These differences were not significant.

## 4. Discussion

The most commonly used combination for conscious sedation in day aesthetic surgeries is midazolam and fentanyl [9]. Midazolam is ideal for conscious sedation because it provides moderate sedation and has an acceptable side-effect profile [10]. Fentanyl is also an ideal analgesic agent for most conscious sedation regimens [10]. Midazolam has been used successfully in both continuous and bolus fashions [11]. The bolus fashion is preferred for blepharoplasty, which has a relatively short operation time and requires patient cooperation (opened eyes) during surgery. However, more cases of respiratory depression and a sudden increase in blood pressure were reported in surgeries involving the bolus fashion compared to continuous infusion [9,12]. These side effects may exacerbate swelling and increase comorbidities in facial aesthetic surgeries.

Dexmedetomidine is a new sedative approved by the United States Food and Drug Administration in 1999 that was recently introduced in outpatient anesthesia in Korea. Dexmedetomidine is a highly selective alpha-2 agonist that has several advantages as a sedative [12]. It secures the airway and respiration even under moderate sedation and carries a low risk of cardiopulmonary instability [13]. Additionally, it inhibits the sympathetic nerve. Dexmedetomidine decreases the amount of analgesic needed during and after surgery, presenting analgesic effects [14]. Notably, there was no difference in the degree of sedation in a comparative study with other sedatives [12,15]; however, dexmedetomidine was the preferred sedative of practitioners because it made facial procedures more comfortable and safe [16]. An elimination half-life of 2.1–3.1 h was reported in healthy volunteers using dexmedetomidine. Pharmacologically, the advantage of a long half-life is that stable and safe sedation is possible. The disadvantage is that early recovery is difficult, and recovery is often late, so it is suitable for surgery of more than 2 h.

Successful surgical treatment depends on the administration of an optimal amount of local anesthesia. Excessive local anesthetics can cause facial swelling, which may interfere with sophisticated facial surgery. Local anesthetics alone are less profound and suffer from the limitation of a short duration of action compared with local anesthetics with adjuvants. The adjuvants are coadministered with local anesthetic agents to improve the onset and/or duration of analgesia, which include both opioids and nonopioids, including epinephrine and an alpha-2 agonist. In the literature [17,18,19], dexmedetomidine blunted pain signals by inhibiting epinephrine release and exhibiting analgesic properties. Pharmacokinetically, continuous infusion better controls intraoperative pain management compared to a bolus injection [20]. In our study, the total amount of local anesthetic was significantly lower (*p* < 0.001) in the dexmedetomidine group despite the longer incision length and surgery duration. Based on these results, the local anesthetic demand was decreased more in the dexmedetomidine group due to analgesic effects.

In our hospital, NSAIDs are usually used for postoperative pain control. As they tend to increase bleeding, increasing NSAID dosages may increase hematoma formation. Lee et al. reported that postoperative pain and two or more doses of ketorolac postoperatively were significantly associated with the risk of postoperative hematoma formation [6]. The perioperative risk factors of hematoma formation in multivariate analysis and the use of ketorolac postoperatively showed more than twice the tendency of hematoma formation than postoperative pain. Moreover, Cawthorn et al. reported that patients who received ketorolac were at an increased risk of requiring surgical re-exploration for hematoma evacuation (relative risk [RR] = 3.6; 95% confidence interval [CI], 1.4–9.6) [7]. In our study, the ketoprofen dose was significantly lower in the dexmedetomidine group than in the conventional group (*p* = 0.028). The VAS Score, which represents the patient’s pain expression in the PACU, was significantly lower in the dexmedetomidine group compared to the conventional group (*p* = 0.004; Table 5). This might be caused by the longer-lasting analgesic effect of dexmedetomidine compared to the conventional sedation method.

Facial hematomas are a possible complication of aesthetic facial surgery [17]. A sudden increase in blood pressure appears to be the single most important cause of hematomas, especially during the postoperative period. Moore et al. found that dexmedetomidine resulted in a significant reduction and maintenance of blood pressure from anesthesia onset until PACU discharge [3]. Dexmedetomidine’s analgesic properties attenuate the hypertensive response and improve hemodynamic stability perioperatively [21]. In the dexmedetomidine group, hypertension events and hematoma formation were decreased simultaneously. This suggests that continuous dexmedetomidine infusion is a more suitable sedation method for perioperative pain control in patients who have undergone blepharoplasty than a midazolam/fentanyl bolus injection. Sedation using dexmedetomidine continuous infusion reduced the incidence of postoperative swelling and hematomas in blepharoplasty patients, resulting in positive effects on surgical outcomes and patient satisfaction.

Dexmedetomidine is known to induce bradycardia as a highly selective alpha-2 agonist. It has been observed that bradycardia (<60 beats/min) had been occasionally experienced during prolonged surgeries when dexmedetomidine was administered. However, since blepharoplasty was a short-duration surgery and the dosage of dexmedetomidine was well controlled during the procedure, severe bradycardia (<50 beats/min) was not observed in this study.

The mechanisms of dexmedetomidine and fentanyl on urinary retention are opposite to each other. Fentanyl has a short duration of action on μ and δ receptors in the spinal cord, reducing sensory input from the urinary tract, decreasing the urge to void and muscle contraction, and increasing bladder capacity. Additionally, this drug reduces the abdominal extract of the spinal nervous system, impairing the regulatory function of internal urethral sphincter relaxation and bladder contraction. On the other hand, dexmedetomidine stimulates α2-adrenergic receptors in the central nervous system to increase urination urge along with muscle relaxation. Ghada et al. reported significant differences in the incidence of urinary retention between the group treated with dexmedetomidine and the group treated with fentanyl [22]. Therefore, the use of dexmedetomidine as a sedative drug may be more effective in preventing postoperative urinary retention. In our study, a lower incidence of urinary catheterization was observed in the dexmedetomidine group compared to the conventional group (Table 5).

Anesthesia-related patient safety has become important to plastic surgeons [23], given recent media attention on medical errors, concerns about day surgery safety, and the increasing number of outpatient aesthetic procedures [24]. Bitar et al. found that the most common sedation-related complications were dyspnea and respiratory depression [23]. The ideal sedative drug in a day surgery does not necessarily induce respiratory depression, but midazolam, fentanyl, and ketamine used in conventional sedation may cause respiratory depression. In our study, severe intraoperative hypoxia was reported in the conventional group, but none of the dexmedetomidine group reported severe hypoxia during sedation. Dexmedetomidine infusion is suitable in facial surgeries such as blepharoplasty.

The occurrence of post-surgical confusion or delirium is an important factor in determining same-day discharge for surgical patients. It is anticipated that the incidence of delirium is very low due to our anesthesia approach involving a combination of light sedation and local anesthesia, as well as the short time of the surgical procedures. In this cohort study, there were no cases where same-day discharge was not feasible due to severe postoperative cognitive impairment. Paul et al. reported that the incidence of postoperative cognitive dysfunction (POCD) was high in major surgeries, with 17% for total hip joint replacement surgery and 43% for coronary artery bypass grafting surgery at postoperative day 7 [25]. However, there has been no specific research on the occurrence rate of cognitive dysfunction, specifically after light sedation, making it difficult to determine the exact incidence rate of POCD. Considering that POCD is influenced by factors such as the surgery itself, the duration of surgery, and the depth of anesthesia, it is anticipated that the occurrence rate of POCD would be very low in this study.

The important thing is that the analgesic effect of dexmedetomidine is higher than that of the conventional sedation method, so in conclusion, the use of ketoprofen in PACU and the total amount of local anesthetic were decreased. However, differences in pain medication use in recovery may be related to the long half-life of dexmedetomidine itself. Therefore, additional research is necessary to find the exact correlation. Contrary to the existing literature, our study had many complaints of nausea and antiemetics administration in the dexmedetomidine group, but the difference was negligible and not statistically significant (nausea; *p* = 0.356, antiemetic drug use; *p* = 0.256; Table 5). Future studies could explore this and the effect it has on postoperative nausea and vomiting. The other limitations of this study include its retrospective nature, which prevents a direct correlation between changes in perioperative parameters and analysis of direct effects on the response of sedative drugs. Therefore, the following limitations were identified: patients were not randomized, blinding was not implemented, no primary outcome was specified, and the study analyzed data from a four-year period, including data from five years ago. Second, in order to evaluate POCD, a more sophisticated and well-controlled approach is necessary, which may be a limitation of cohort studies. Therefore, we intend to conduct prospective research in the future. Third, we were unable to determine whether dexmedetomidine actually increased postoperative urinary retention. Further investigation is needed to compare the different physiology of urinary retention between the dexmedetomidine group and the benzodiazepine/opioid group.

## 5. Conclusions

In this study, dexmedetomidine infusion was associated with more hemodynamic stability and postoperative analgesia than midazolam and fentanyl bolus injection during sedation in blepharoplasty patients. NSAIDS consumption and hypertensive events were lower in the dexmedetomidine group. Sedation with dexmedetomidine continuous infusion is associated with less hematoma formation and postoperative bleeding than sedation with midazolam and fentanyl. Dexmedetomidine could secure self-respiration during sedation. Dexmedetomidine infusion may be a good alternate sedative method for blepharoplasty.

## Figures and Tables

**Table 1:** Demographic characteristics of the patients.

		Dexmedetomidine Group (n = 113)	Conventional Group (n = 137)	Total (n = 250)	*p*-Value
Age (years)		57.9 ± 7.6	57.6 ± 12.2	57.8 ± 10.4	0.817
Sex	Male (%)	43 (38.1)	46 (33.6)	89 (35.6)	0.462
	Female (%)	70 (61.9)	91 (66.4)	161 (64.4)	
ASA class	I	11 (9.7)	21 (15.3)	32 (12.8)	0.188
	II	102 (90.3)	116 (84.7)	218 (87.2)	
BMI (kg/m^2^)		24.1 ± 2.5	24.6 ± 3.3	24.4 ± 3.0	0.159

Values are presented as mean ± standard deviation. Abbreviations: ASA, American Society of Anesthesiologists; BMI, Body Mass Index.

**Table 2:** Characteristics of sedation methods according to the year.

	Dexmedetomidine Group (n = 113)	Conventional Group (n = 137)	*p*-Value
2018 (Year)	23 (52.3)	21 (47.7)	0.517
2019 (Year)	26 (44.1)	33 (55.9)	
2020 (Year)	34 (50.0)	34 (50.0)	
2021 (Year)	22 (38.6)	35 (61.4)	
2022 (Year)	8 (36.4)	14 (63.6)	

**Table 3:** Characteristics of the surgery-related profiles.

		Dexmedetomidine Group (n = 113)	Conventional Group (n = 137)	*p*-Value
Total local anesthetic (cc)		5.6 ± 2.3	7.4 ± 2.3	<0.001 *
Incision	Total length (cm)	8.3 ± 0.9	8.2 ± 0.9	0.202
	Number: two (%) **	113 (100.0)	137 (100.0)	1.000
Duration of operation (min)		96.0 ± 30.3	94.0 ± 29.0	0.605

Values are presented as mean ± standard deviation. * Statistically significant. ** The number of incisions was two in both groups.

**Table 4:** Characteristics of complication-related profiles.

		Dexmedetomidine Group (n = 113)	Conventional Group (n = 137)	*p*-Value
Hypoxia episode (%)		3 (2.7)	28 (20.4)	0.001 *
Hypoxia severity	Normal (%)	110 (97.3)	109 (79.6)	<0.001 *
	Mild/Moderate (%)	3 (2.7)	26 (19.0)	<0.001 *
	Severe (%)	0 (0)	2 (1.4)	0.503
Hematoma (%)	Major	0 (0)	1 (0.7)	1.000
	Minor	2 (1.8)	14 (10.2)	0.007 *
BP change	Hypertension	4 (3.5)	20 (14.6)	0.003 *
	Hypotension	7 (6.2)	4 (2.9)	0.231

Values are presented as mean ± standard deviation. Hypoxia severity was graded as follows: (a) normal: 95–100% SpO_2_; (b) mild hypoxia: 91–94% SpO_2_; (c) moderate hypoxia: 86–90% SpO_2_; and (d) severe hypoxia: ≤85% SpO_2_. Major hematomas were defined as those requiring open evacuation and coagulation of the offending bleeders, while minor hematomas were defined as those that resorbed with a massage or spontaneously without treatment. Abbreviations: BP, Blood pressure. * Statistically significant.

**Table 5:** Characteristics of the recovery-related profiles.

		Dexmedetomidine Group (n = 113)	Conventional Group (n = 137)	*p*-Value
Duration of PACU stay (min)		110.5 ± 30.7	84.3 ± 41.4	<0.001 *
Pain	VAS Score	1.0 ± 0.4	1.2 ± 0.8	0.004 *
	Ketoprofen use (%)	3 (2.7)	13 (9.5)	0.028 *
Nausea	Complain (%)	12 (10.6)	10 (7.3)	0.356
	Antiemetic drug use (%)	12 (10.6)	9 (6.6)	0.251
Urinary catheterization (%)		2 (1.8)	6 (4.4)	0.300

Values are presented as mean ± standard deviation. The pain score consists of two end-points representing 0 (no pain) and 10 (maximal pain). Ketoprofen was used to control postoperative pain in the PACU. Abbreviations: PACU, post-anesthesia care unit; VAS, visual analog scale. * Statistically significant.

## Data Availability

Not applicable.
